# Differential proteomics profile of microcapillary networks in response to sound pattern-driven local cell density enhancement

**DOI:** 10.1016/j.bbiosy.2024.100094

**Published:** 2024-03-29

**Authors:** N. Di Marzio, R. Tognato, E. Della Bella, V. De Giorgis, M. Manfredi, A. Cochis, M. Alini, T. Serra

**Affiliations:** a AO Research Institute Davos, 7270 Davos, Switzerland; b Department of Health Sciences, Center for Translational Research and Autoimmune and Allergic Diseases (CAAD), University of Piemonte Orientale, 28100 Novara, Italy; c Department of Translational Medicine, Center for Translational Research and Autoimmune and Allergic Diseases (CAAD), University of Piemonte Orientale, 28100 Novara, Italy; d CTR Department, MERLN Institute for Technology-Inspired Regenerative Medicine, Maastricht University, Maastricht, the Netherlands; e Collaborative Research Partner, AO CMF CPP Bone Regeneration, Davos, Switzerland

**Keywords:** Bio-assembly, Sound patterning, Microcapillary networks, Cell density enhancement, Proteomic analysis

## Abstract

Spatial cell organization and biofabrication of microcapillary networks in vitro has a great potential in tissue engineering and regenerative medicine. This study explores the impact of local cell density enhancement achieved through an innovative sound-based patterning on microcapillary networks formation and their proteomic profile. Human umbilical vein endothelial cells (HUVEC) and human pericytes from placenta (hPC-PL) were mixed in a fibrin suspension. The mild effect of sound-induced hydrodynamic forces condensed cells into architected geometries showing good fidelity to the numerical simulation of the physical process. Local cell density increased significantly within the patterned areas and the capillary-like structures formed following the cell density gradient. Over five days, these patterns were well-maintained, resulting in concentric circles and honeycomb-like structures.

Proteomic analysis of the pre-condensed cells cultured for 5 days, revealed over 900 differentially expressed proteins when cells were preassembled through mild-hydrodynamic forces. Gene ontology (GO) enrichment analysis identified cellular components, molecular functions, and biological processes that were up- and down-regulated, providing insights regarding molecular processes influenced by the local density enhancement. Furthermore, we employed Ingenuity Pathway Analysis (IPA) to identify altered pathways and predict upstream regulators. Notably, VEGF-A emerged as one of the most prominent upstream regulators.

Accordingly, this study initiates the unraveling of the changes in microcapillary networks at both molecular and proteins level induced by cell condensation obtained through sound patterning. The findings provide valuable insights for further investigation into sound patterning as a biofabrication technique for creating more complex microcapillary networks and advancing in vitro models.

## Highlights

• Effective spatial organization of endothelials and pericytes by sound patterning.
• Radiation forces increase local cell density, forming continuous capillary-like structures.
• Molecular changes post cell condensation verified by 900+ regulated proteins.
• Cell communication and remodeling modulation due to cellular condensation.
• Ingenuity pathway analysis highlights VEGF-A's as upstream regulator.

## Graphical abstract

**Figure:**
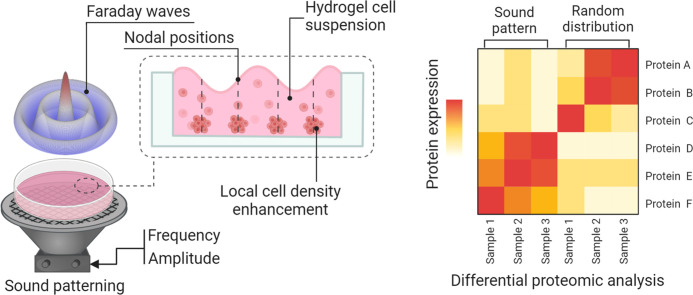

## 1 Introduction

Human tissues consist of densely packed cells, with concentrations varying depending on the tissue physiology. Some tissues may have cell concentrations exceeding tens of millions of cells per milliliter (1 to 3 billion cell mL^−1^) [1]. In the field of tissue engineering and regenerative medicine, recapitulating the native cell packing densities is under continuous investigation [2,3]. As an example, in cardiac tissue engineering a cell density greater than 40 million cell mL^−1^ is often required to enable spontaneous contraction of the tissue [4]. In cartilage tissue engineering, high seeding cell density (15 to 90 × 10^6^ cell mL^−1^) results in constructs with biomechanical properties close to the native cartilage [5]. A densely packed cellular environment is especially crucial during vasculogenesis, the process of *de novo* blood vessel formation. Vasculogenesis [6] involves the initial differentiation of endothelial precursor cells (angioblasts) and their assembly into a densely packed primitive vascular plexus. This initial remodeling lays the foundations for the development of more complex vascular structures that occurs in the next phase of angiogenesis. It has been shown that a percolative transition exists, which can lead or not to the formation of capillary networks depending on cell density [7]. With the development of more complex in vitro models, the native vasculature of the modeled tissues should be included to capture the complete tissue physiology as well as to avoid necrosis due to the lack of a proper vascularization [8]. It is widely acknowledged that replicating the vascular component in vitro is crucial, whether the goal is to fabricate large-scale (mm to cm) 3D biological constructs or to create 3D models for high throughput drug screening applications. The biofabrication of large-scale constructs is hindered by the development of a necrotic core due to the limited diffusion of nutrients for cells, which can only penetrate approximately 200 µm into 3D structures [9]. Moreover, when in vitro models are designed for drug screening applications, incorporating the vascular component leads to more reliable readouts as well as to study new strategies aimed at ameliorate the biodistribution of the active principle [10].

For the biofabrication of capillary networks, Endothelial cells (ECs) and stromal or perivascular cells are commonly used. ECs assume a central role in vasculogenesis and angiogenesis, as they form the innermost lining of blood vessels. They possess the remarkable ability to self-assemble into tubular structures and create a basal lamina, providing a structural matrix to which other cells adhere during the maturation of the vascular tissue [11]. In smaller capillaries, perivascular cells, such as pericytes, play a pivotal role in supporting angiogenesis and contributing to tissue regeneration, wound healing, immune regulation, and modulation of neuroinflammation [12]. Importantly, the molecular milieu produced by pericytes, often referred to as the secretome, is tissue-specific and rich in biochemical factors like growth factors, anti-inflammatory molecules, microvesicles, and extracellular matrix components [13].

The isolation and in vitro culture of ECs and perivascular cells from human tissues have paved the way for researchers to explore their behavior and interactions. Human Umbilical Vein Endothelial Cells (HUVEC) have been vastly used for engineering microcapillary networks in vitro [14]. Under appropriate conditions, these cells can spontaneously form tubular structures resembling primitive capillary vessels. The expression of specific markers, such as vascular-endothelial cadherin (VE-cadherin), is indicative of the formation of tight junctions between ECs, confirming the tissue maturation and functionality in vitro. Several approaches have been explored to replicate vasculogenesis within in vitro models, involving the co-culture of ECs and pericyte cells, allowing them to self-assemble into vascular-like structures [15].

Biofabrication technologies used to meet the critical needs of high cell density [3,16] and spatial organization in vitro can generally be categorized based on whether they involve direct contact with the cells or are contactless. In direct contact methods such as 3D bioprinting, augmenting cell density in the bioink would lead to a rise in shear stress at the dispensing nozzle. While this issue could be alleviated by enlarging the nozzle's diameter, such change would affect the overall printing resolution [17,18]. Alternatively, robotic arm can singularly pick cellular aggregates through suction and deposit at the desired site [19]. Nonetheless, the fabrication process can be time-consuming, especially for larger-scale constructs.

In turns, contactless methods as external fields such as light [20], magnetic fields [21,22], and acoustic fields [23] results in faster printing time compared to the ones using direct contact. The drag forces generated within fluids by traveling acoustic waves have been exploited for condensation of cells and particles in a liquid phase [23]. Sound patterning (also referred as acoustic bioassembly) has emerged as a valuable method for assembling densely packed cell patterns, ultimately giving rise to networks of capillary-like structures with predetermined morphologies [24,25]. Sound patterning harnesses low-frequency waves to generate hydrodynamic drag forces, enabling the precise assembly of cells or cell aggregates within fluids [25]. This contactless and cell-friendly process enhances cell-cell contacts, cross-talk, and anisotropic organization. A large literature have previously demonstrated the potential of the local cell density enhancement induced by sound patterning for engineering closely packed organotypic constructs such as in vitro models of hepatic lobules [26], of nerve ingrowth related to low back pain [27], of brain tissue [28] and 3D osteoinductive constructs [29], are among the most recent breakthrough in the sound pattering field.

Despite our previous results in forming continuous and hierarchically organized capillary networks after sound patterning [24,25], the molecular and protein regulation influenced by the augmented cell concentration remain to be fully elucidated.

In this study we used proteomic analysis to clarify the changes induced by enhancing the local cell concentration within a natural extracellular matrix environment of a fibrin gel colonized by a 3D architecture of cells. The cell density enhancement was designed to mimic capillary like structures obtained by on demand morphological organization. The cytocompatibility and user-friendly approach demonstrated by the innovative sound patterning technology here presented, proved this approach as a very promising biofabrication strategy for studying functional cell condensation.

## 2 Material and methods

All chemicals were purchased from Sigma-Aldrich, Switzerland, and used as received without any further purification unless stated differently. Cell culture flasks and plastic ware were purchased from Techno Plastic Products AG (TPP, Trasadingen, Switzerland). Cell culture media and supplements related to cell culture were from Gibco (Thermo Fisher Scientific, Basel, Switzerland).

### 2.1 Fibrin gel preparation

Fibrinogen stock solution, consisting of 64 % protein with a clottable fraction of 90 %, was prepared at a concentration of 15 mg mL^−1^ in phosphate-buffered saline (PBS). This solution was incubated at 37 °C for 1 h. The solution was passed through sterile 0.2 μm pore filters, aliquoted, and stored at −20 °C. A thrombin stock solution with a concentration of 100 IU mL^−1^ was prepared by reconstituting lyophilized thrombin sourced from human plasma. The reconstitution was performed in a solution containing 1.1 % (w/v) NaCl and 2 mM CaCl_2_, and the resulting solution was stored at −20 °C.

To create the fibrin gel, working solutions of fibrinogen were prepared by diluting the stock solution to a final concentration of 5 mg mL^−1^ in a 1.1 % (w/v) NaCl solution. Similarly, the thrombin working solution was prepared by diluting the stock solution to a final concentration of 0.5 IU mL^−1^ in EGM-2 medium. Equal volumes of the fibrinogen and thrombin working solutions were mixed on ice, resulting in a final concentration of 2.5 mg mL^−1^ fibrinogen and 0.25 IU mL^−1^ thrombin.

### 2.2 HUVEC and pericyte cells expansion

Green Fluorescent Protein-expressing Human Umbilical Vein Endothelial Cells (GFP-HUVEC, cAP-0001GFP) were purchased from Angio-Proteomie, Boston, USA, and expanded in Complete Endothelial Cell Growth Medium (EGM-2, Lonza Group AP, Switzerland) in pre-coated culture flasks (Quick Coating Solution, Angio-Proteomie). Human pericytes from placenta (hPC-PL, PromoCell GmbH, Heidelberg, Germany) were expanded in complete Pericyte Growth Medium 2 (PromoCell GmbH). For both cell types, cell culture media were refreshed every 48 h, cells were passaged by trypsinization at 90 % confluency. Cell culture was maintained at 37 °C in a 95 % humidified incubator at 5 % CO_2_.

### 2.3 Microcapillary sound patterning

The system, known as Sound Induced Morphogenesis (SIM) device (CymatiX, mimiX Biotherapeutics, Biel/Bienne, Switzerland) [24,25], was employed to generate the sound patterned cells which self-assembled into microcapillaries. The SIM device consists of a mechanical driver that vibrates vertically, a sample holder, and a control system that actuate the mechanical driver with specific frequency and amplitude. The desired cell-laden hydrogel precursor is loaded into the patterning chamber and vibrated vertically.

A custom-made chamber was prepared for the patterning procedure. Round discs fitting with a 60 mm petri dish were obtained from a 1 mm thick poly(methyl methacrylate) (PMMA) sheet. Round and square geometries (21 mm in diameter and 15 mm in length respectively) were cut out from the PMMA discs and the obtained inserts were bounded to the petri dish with medical double adhesive tape (3 M Science. Applied to Life™, Saint Paul, USA) forming the sound patterning chamber which underwent UV sterilization for 1 h. At day 0 GFP-HUVEC and hPC-PL cells were collected and mixed in a cell suspension at a ratio of 10:1 HUVEC:PC-PL. This mixed cell suspension was used to create a thrombin-EGM-2-cells suspension (3 × 10^6^ cells mL^−1^) which was kept on ice until further use. On ice, fibrinogen (5 mg mL^−1^) and thrombin-EGM-2-cells suspension were mixed at 1:1 volume ratio and 311 µl (or 200 µl) were loaded into the round (or square) patterning chamber. The patterning chamber was transferred to the sample holder. By applying a sound vibration with a characteristic frequency of 54 Hz and vertical acceleration of 1.2 g, the concentric ring (or honeycomb-like) patterns were obtained within 2 min from the start of the sound vibration. After fabrication, the patterning chamber was left on the vibration plate for additional 2 min to allow for fibrin gelation and pattern stabilization. Subsequently, the patterning chamber was incubated at 37 °C for 5–10 min to further facilitate gelation of the fibrin gel. The patterning chamber was then filled with 4 ml of EGM-2 growth medium and cultured at 37 °C, 5 % CO_2_ humidified atmosphere up to 5 days. The same cell suspension of HUVEC:hPC-PL in fibrin was also seeded into the patterning chamber without sound application to promote the formation of a randomly organized capillary network.

### 2.4 Imaging of the microcapillary networks over time

Microcapillary networks were visually checked by confocal microscope from day 0 (pattern formation) until day 5 (end point) and image analysis approaches were used to characterized capillary networks growth. At day 0, fluorescence images of the condensed cell patterned lines or randomly distributed cells, were acquired with an Olympus CK40 inverted microscope (Olympus Corporation, Tokyo, Japan) equipped with 4× and 10× magnification objectives and later used to quantify the GFP+ area covered by the HUVEC after sound patterning. At day 1, 3, and 5, complete overview images of patterned and random samples were acquired with a Zeiss LSM800 confocal microscope equipped with a CCD camera (Axiocam 506 color) and 5× magnification objective (Zeiss, Oberkochen, Germany). A scanning area of 543 mm^2^ (or 350 mm^2^) was defined for the circular (or square) geometry, a 350 μm thick Z-stack with a slice's thickness of 30 μm was defined for imaging the volume occupied by the capillaries, 190 mm^3^ (or 122.5 mm^3^). Detail images of patterned and random capillaries were acquired with 10× magnification objective, scanning area of 1.3 mm^2^ and 71.41 μm thick Z-stack with a slice's thickness of 1.9 μm (total scanning volume of 0.12 mm^3^). From the Z-stacks, the maximum intensity Z-projections of the images were generated in ImageJ software (NIH, Bethesda, MD, USA) and used for image analysis. From the sample overview images the Radial Profile signal was extracted to monitor the network evolution over time. From the details images the local capillary thickness was analyzed.

### 2.5 Numerical simulation of the liquid surfaced displacement during sound patterning

The surface fluid displacements for circular chamber have been simulated using Bessel's function of the first kind. Considering the (m,n) vibrating modes, the Bessel's equation [30] reads:

$$\zeta =\;J_{m}\left(\frac{\alpha_{mn}}{R}r\right)\cos\left(m\theta \right)$$

where $J_{m}\left(\cdot \right)$ is the Bessel function of the first kind of orderm, $\alpha_{mn}$ is the *n*th zero of $J_{m}\left(\cdot \right)$, θ and the r are the radial and azimuthal co-ordinate and R is the diameter of the chamber. In the specific case we used *R* = 21 mm, *m* = 0, *n* = 4. The surface deformation for a squared chamber were simulated following the superposition on of two vibrating modes (*m* = 5, *n* = 1) according to the following equation [31]:

$$\zeta =\;\left(\text{cos}\left(\frac{n\pi }{l_{x}}x\right)\text{cos}\left(\frac{m\pi }{l_{y}}y\right)-\text{cos}\left(\frac{m\pi }{l_{x}}x\right)\text{cos}\left(\frac{n\pi }{l_{y}}y\right)\right)$$

where $l_{x}$ and $l_{y}$ are the chamber dimensions of 15 mm. Numerical simulations were carried out using Python Version 3.9, Numpy and Matplotlib library on a personal computer equipped with a processor 11th Gen Intel(R) i5-1145G7 and 16 GB of RAM.

### 2.6 Image analysis and morphological characterization

The evolution of the microcapillaries pattern was monitored over a 5-day culture period by measuring the radial intensity profile of the concentric rings pattern [32]. The peaks of the intensity profile corresponded to individual circles.

The valley-to-peak ratio of the radial profile's signal was measured for each day over the culture period, as well as the thickness of the rings. The latter was calculated based on the Full Width at Half Maximum (FWHM) of the pattern's line peak in the radial intensity profile signal [29].

The local cell density enhancement was assessed using images captured immediately after sound patterning (day 0). Square Regions of Interest (ROIs) were defined with side lengths equal to the width of the pattern lines. These ROIs were used to quantify the GFP+ area at various locations within the images. A binary mask was generated from the segmented images and, for each location, three ROIs were placed. The specific locations analyzed were as follows:

1. 'On pattern': ROIs positioned directly on the patterned line.
2. 'Distal': ROIs positioned in the area adjacent to the patterned line towards the periphery of the pattern.
3. 'Proximal': ROIs positioned in the area adjacent to the patterned line towards the center of the pattern.
4. 'Random': ROIs positioned on a region representing random cell distribution under stationary conditions.

We conducted these quantifications for both the first and second rings of the concentric rings pattern.

The local thickness analysis [33] of the microcapillary network was conducted on representative images of patterned capillary structures and randomly assembled capillaries at day 1, 3, and 5. After generating binary masks of the images, the Local Thickness function from ImageJ was employed to extract a local thickness mask in which pixels belonging to areas of the same thickness were assigned identical values in microns. Subsequently, histograms of these local thickness masks were generated.

To analyze the general orientation presented by capillary-like structures in overview images, the Fast Fourier Transform (FFT) of the sample overview image was obtained using ImageJ. The high-frequency components were then segmented, and the Inverse FFT was calculated using ImageJ's dedicated function. The OrientationJ Distribution plugin of ImageJ was utilized to extract the orientation distribution of the structures in the images. Within the plugin, the Cubic Split Gradient was applied for the structure tensor, using a Gaussian window (*σ*) of 4 pixels and a minimum energy threshold of 30 %. The angle distribution data were subsequently smoothened, normalized, and presented in a polar plot.

Statistical analysis was performed in GraphPad Prism 8.0.2 for Windows (Dotmatics, Boston, USA) using a one-way ANOVA test to determine significant differences between multiple conditions.

### 2.7 Immunostainings

At Day 5, the samples were washed in PBS and fixed in paraformaldehyde 4 % for 1 h at room temperature (RT). The non-specific binding sites were blocked with 4 % BSA and permeabilization was performed with Triton X-100 2 % in PBS for 30 min. Anti-Hu CD-144 (VE-cadherin) PE-conjugated clone 16B1 (Invitrogen, Thermo Fisher) 1:100 solution in PBS was added for 1 h at room temperature. After washing with PBS, cell nuclei were counterstained with 4′,6-diamidino-2-phenylindole (DAPI) (1:800) and co-visualized with a Zeiss LSM800 confocal microscope.

### 2.8 Sample collection and peptides extraction

At day 5 the fibrin gels containing the sound patterned or random samples were washed with PBS and incubated at 37 °C with 1 ml of 2 mg mL^−1^ Nattokinase solution prepared in PBS 1 mM EDTA [34] (Fig. S1, b). Samples were collected in Eppendorf tubes, centrifuged at 400 g, 4 °C, 5 min and the supernatant were discarded. The pellets were washed with cold (4 °C) PBS and centrifuged at 400 g, 4 °C, 5 min. The supernatant was discarded, and the samples were snap frozen and stored at −80 °C until further processing. Cellular pellets were lysed with Radioimmunoprecipitation Assay (RIPA) buffer and sonicated. Proteins were precipitated with cold acetone and resuspended. Proteins were then reduced in 25 µL of 100 mM NH_4_HCO_3_ with 2.5 μL of 200 mM DTT (Merck, Darmstadt, Germania) at 60 °C for 45 min and next alkylated with 10 μL 200 mM iodoacetamide (Merck) for 1 h at RT in dark. Iodoacetamide excess was removed by the addition of 200 mM DTT. Digested peptides were dried by Speed Vacuum, desalted, and analyzed on an Ultimate 3000 RSLC nano coupled directly to an Orbitrap Exploris 480 with a High-Field Asymmetric Waveform Ion Mobility Spectrometry System (FAIMSpro) (all Thermo Fisher Scientific). Samples were injected onto a reversed-phase C18 column (15 cm × 75 µm i.d., Thermo Fisher Scientific) and eluted with a gradient of 6 % to 95 % mobile phase B over 80 min by applying a flow rate of 300 nL/min, followed by an equilibration with 6 % mobile phase B for 8 min. Mass spectrometry (MS) scans were performed in the range of *m/z* 375–1200 at a resolution of 120.000 (at *m/z* = 200). MS/MS scans were performed choosing a resolution of 15.000; normalized collision energy of 30 %; isolation window of 2 *m/z*; and dynamic exclusion of 45 n s. Two different FAIMS compensation voltages were applied (−45 V and −60 V), with a cycle time of 1.5 s per voltage. FAIMS was operated in standard resolution mode with a static carrier gas flow of 4.6 L/min.

### 2.9 Proteomics analysis

The acquired raw MS data files were processed and analyzed using Proteome Discoverer with Chimerys (v3.0.0.757, Thermo Fisher Scientific). SequestHT was used as a search engine and the following parameters were chosen. Database: Homo sapiens (Uniprot, downloaded on 01-02-2018) enzyme: trypsin; max. missed cleavage sites: 2; static modifications: carbamidomethyl (C); dynamic modifications: oxidation (M); precursor mass tolerance: 10 ppm; fragment mass tolerance: 0.02 Da. Only peptides and proteins with FDR value <0.01 were reported. Abundance of identified peptides was determined by label-free quantification (LFQ) using match between runs. Statistical analyses and *t*-test were performed on protein abundances using MetaboAnalyst software [35] (accessed at the web site: https://www.metaboanalyst.ca/). Principal component analysis (PCA) was performed using protein abundances in pattern and random samples through MetaboAnalyst software [35] (accessed at the web site: https://www.metaboanalyst.ca/). The scores from the first and the second PCs were used to plot the samples. Modulated proteins were analyzed through Database for Annotation, Visualization and Integrated Discovery (DAVID) (version 6.8) (available at the web site http://david.abcc.ncifcrf.gov/) and Ingenuity Pathway Analysis (IPA, QIAGEN, Hilden, Germania). The differentially regulated pathways were grouped based on their relevance in the presented in vitro tissue engineering application, therefore 3 categories were highlighted: “Angiogenesis and Remodeling”, “Intercellullar Communication” and “Stress Response”. Lastly, the set of significant differentially expressed proteins was manually curated. The most relevant proteins, contextual to the experimental conditions, were clustered together according to their known functions and represented as a bubble plot.

## 3 Results

### 3.1 Experimental outcomes match with numerical simulation of the sound patterning process

**Fig. 1:**
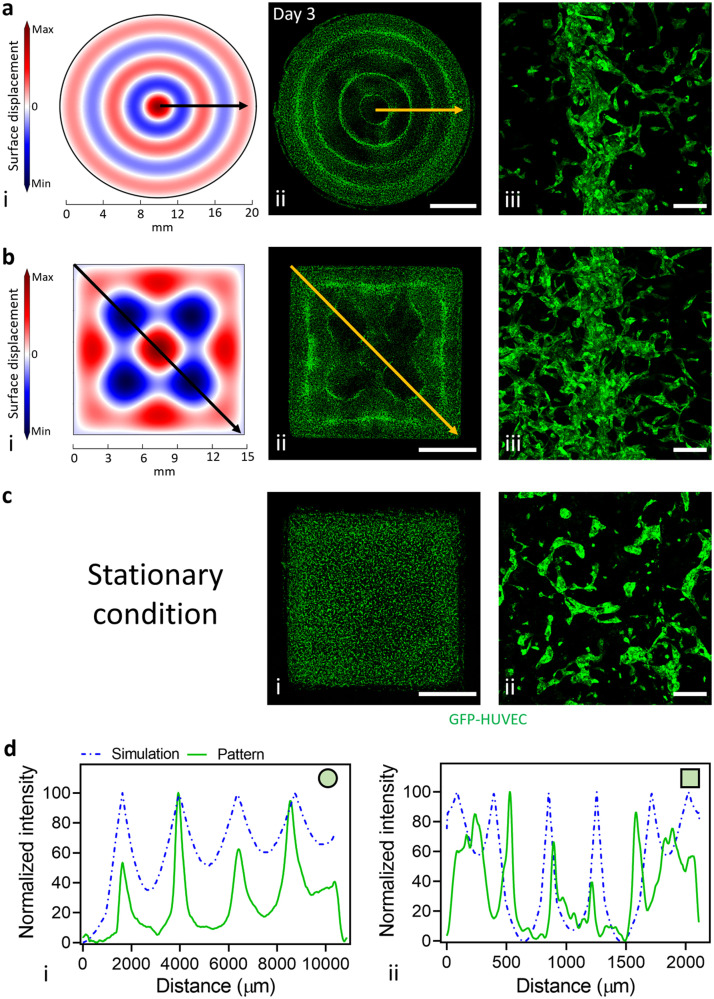
Computational model of sound-induced liquid surface displacement and prepatterned microcapillary networks in round and square geometries. The numerical simulation of the fluid interface deformation, driven by Faraday waves in (a, i) round and (b, i) square patterning chambers, predicted the final patterned cell configurations. Three days after sound patterning, the condensed cells maintained the prepatterned organization in (a, ii) concentric circles or (b, ii) honeycomb-like shapes. In contrast, under stationary conditions (c, i), a distinct morphological organization could not be achieved (scale bars = 5 mm). GFP-HUVEC and hPC-PL (a&b, iii) self-assembled into hierarchical capillary-like structures following the condensed cell gradient, whereas (c, ii) homogeneously distributed capillary-like structures were obtained in stationary conditions (scale bars = 200 µm). Image analysis of the intensity profile (arrow lines) of the numerical simulation (d) demonstrated the comparable location of the predicted nodal positions and the actual condensed cells distributed within the (i) round and (ii) square geometries.

Numerical simulations of the acoustofluidic phenomenon in sound patterning can be used to verify how particles in the system have been arranged in response to specified boundary conditions. Experimental outcomes in this study were validated from numerical simulations of the sound patterning process. The simulations computed the fluid interface deformation driven in both round (Fig. 1a, i) and square (Fig. 1b, i) patterning chambers. Here, the distribution of the nodal locations reflects the locations with a zero resultant force acting on the particles where cells will eventually accumulate. Hydrodynamic forces guided the accumulation of cells in condensates with distinctive morphologies, either as concentric circles (Fig. 1a, ii) or honeycomb-like shapes (Fig. 1b,ii) depending on the geometry of the chamber. Contrariwise, in the experimental control kept in stationary condition, large cells condensates were not present (Fig. 1c, i). After 3 days of culture, GFP-HUVEC and hPC-PL self-assembled into hierarchical capillary-like structures (Fig. 1a and b, iii) following the gradient of condensed cell. This was not observable with the control condition, where homogeneously distributed capillary-like structures were observed (Fig. 1c, ii). Image analysis of the intensity profile along symmetry lines of the patterns supported these findings, highlighting the consistency between the calculated nodal positions and the actual condensed cells distribution within both round and square geometries (Fig. 1d).

### 3.2 Local cell density enhancement on the patterned lines

**Fig. 2:**
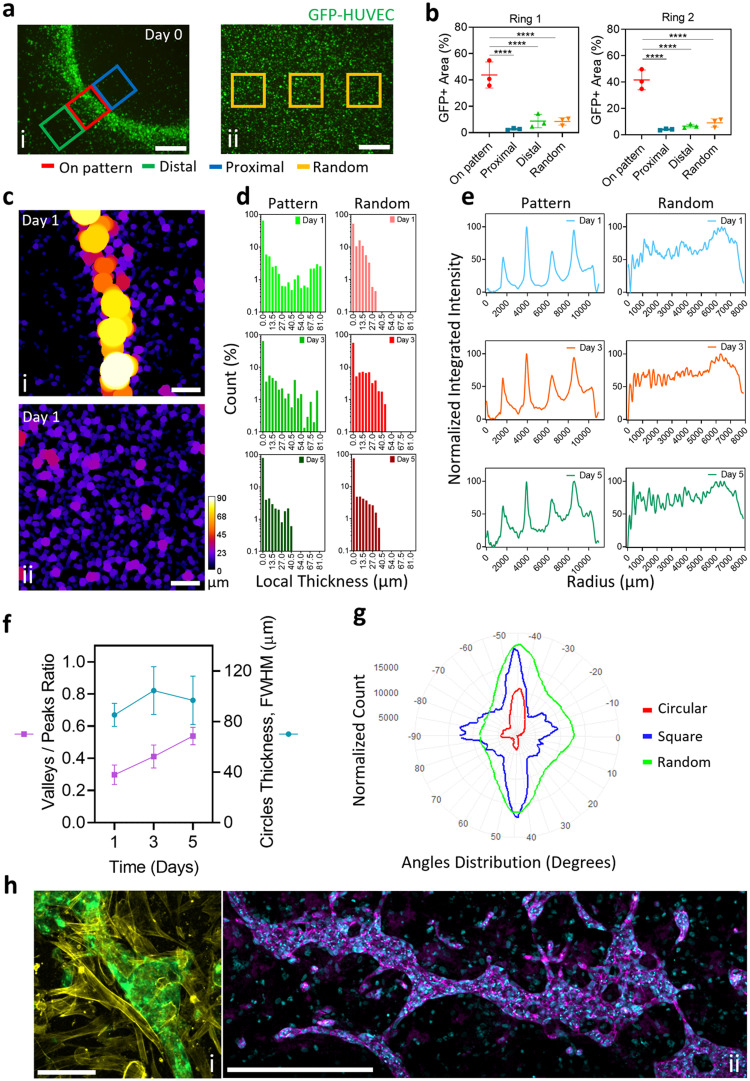
Image analysis-based macroscopic characterization of sound patterned microcapillary networks. Local cell density enhancement after sound patterning was quantified by (a) measuring the GFP+ area of the ROIs placed on (i) the patterned line (“On pattern” ROIs) of the concentric circles pattern and compared with the areas adjacent to the pattern line or with (ii) the cells seeded in stationary condition (“Distal”, “Proximal”, and “Random” ROIs) (scale bars = 500 µm). The measurements (b) were conducted on the first and second rings of the concentric rings pattern and showed 438 ± 103 % and 386 ± 104 % enhancement of local cell density for the first and second rings, respectively, compared to the stationary condition (*n* = 3, mean ± SD, ****p-value <0.0001). The microcapillary network's local thickness analysis (c) was performed on representative images obtained from the concentric circles pattern. Specifically, on (i) the patterned line and on representative images of (ii) the stationary condition (random) at day 1, 3, and 5 of tissue maturation (scale bars = 200 µm). The local thickness results (d) for the patterned capillaries during the 5 days in culture were 13.1 ± 24.7 µm, 10.6 ± 18.9 µm, and 4.1 ± 9.5 µm, whereas 6.6 ± 7.8 µm, 9.3 ± 12.5 µm, and 4.5 ± 8.8 µm for the random distribution in stationary conditions, measured at day 1, 3, and 5, respectively (mean ± SD). The evolution of the microcapillaries pattern (e) was monitored over the 5 days in culture by measuring the radial intensity profile of the concentric rings pattern, with peaks corresponding to single circles on each of the imaging days. The valley-to-peak ratio (f) of the radial profile's peaks was measured over the culture time and showed a linear increase, suggesting capillary growth also in between the concentric rings. Conversely, the thickness of the rings remained relatively constant, ranging between 80 and 120 µm. Analysis of the general orientation (g) presented by capillary-like structures, performed on overview images of samples, revealed that different pattern geometries (round and square) induced different orientation of the capillary-like structures at the macroscopic level. Immunofluorescent staining (h) of the microcapillary networks showed signs of tissue maturation and functionality (representative image from concentric rings pattern). (i) Pericyte cells were found to support the capillary structures (green = GFP-HUVEC, yellow = F-actin, scale bar = 100 µm), and (ii) VE-cadherin was expressed at cell-cell junctions along the patterned capillaries (violet = VE-cadherin, blue = Nuclei, scale bar = 500 µm).(For interpretation of the references to color in this figure legend, the reader is referred to the web version of this article.)

The area percentage covered by cells was quantified on the patterned lines of the concentric circles patterns (Fig. 2a) and compared with the values on the control images obtained after seeding the cells in static conditions. The relative percentage increase in local cell density compared to control was then calculated. A local statistically significant cell density enhancement of 438 ± 103 % and 386 ± 104 % (*n* = 3, mean ± SD) was observed over the first and second rings of the concentric pattern, respectively (Fig. 2b, ****p-value <0.0001). This finding highlights the capacity of sound patterning to significantly increase cell density at specific locations, even when the initial seeding density is maintained at a low level (1.5 × 10^6^ cells mL^−1^). This enhancement can be hypothesized properly mimic the high packing density characteristic of cells in the native tissues.

### 3.3 Microcapillary networks preserve the prepatterned morphologies over 5 days in culture

Additionally, the evolution of the microcapillary patterns was monitored over five days in culture by assessing the radial intensity profile of the concentric rings pattern (Fig. 2e). A linear increase in the valley-to-peak ratio of the radial profile's peaks was observed (Fig. 2f). This indicated capillary growth, also between the concentric rings. On the contrary, the rings’ thickness remained relatively constant, ranging between 80 and 120 µm (Fig. 2f). Moreover, the analysis of the overall orientation of the capillary-like structures revealed that different pattern geometries resulted in distinct macroscopic orientations. Specifically, within the concentric circles pattern, the main angle distribution was centered around −45°, with fewer occurrences at −90° and 45°. In contrast, the honeycomb-like pattern exhibited primary angles at −45°, −90°, and 45°, with fewer occurrences at 0°. Conversely, the randomly formed capillaries displayed a wider and less defined angle distribution (Fig. 2g).

### 3.4 Local remodeling of the cellular clusters into capillaries-like structures with hierarchical thickness after sound patterning

An interesting finding of this study regarded the local remodeling of the patterned cells. The endothelial and pericytes cells self-assembled in capillary-like structures obtained after periodical clustering the cells. After three days tissue maturation, the endothelial cells self-assemble in cells clusters with increasing thickness. At this stage, both thicker and thinner capillary-like structures coexisted, with the thinner structures being more prevalent (Fig. 2c, d). Additionally, the average thicknesses at day 1, 3, and 5 were 13.1 ± 24.7 µm, 10.6 ± 18.9 µm, and 4.1 ± 9.5 µm (mean ± SD) for patterned samples, compared to 6.6 ± 7.8 µm, 9.3 ± 12.5 µm, and 4.5 ± 8.8 µm for the control sample (Fig. S2).

### 3.5 Pericyte support and VE-cadherin expression in the fabricated capillary-like networks

Immunofluorescent staining (Fig. 2h) of the endothelial and pericytes self-assembled networks provided insights into the physiology of the newly-formed capillary-like structures. Pericyte cells were found to support the endothelial structures (Figs. 2h, i & S4), and VE-cadherin was expressed at HUVEC cell-cell junctions (Fig. 2h,ii).

### 3.6 Proteomic profiling of the condensed capillaries revealed over 900 differentially expressed proteins compared to static controls

**Fig. 3:**
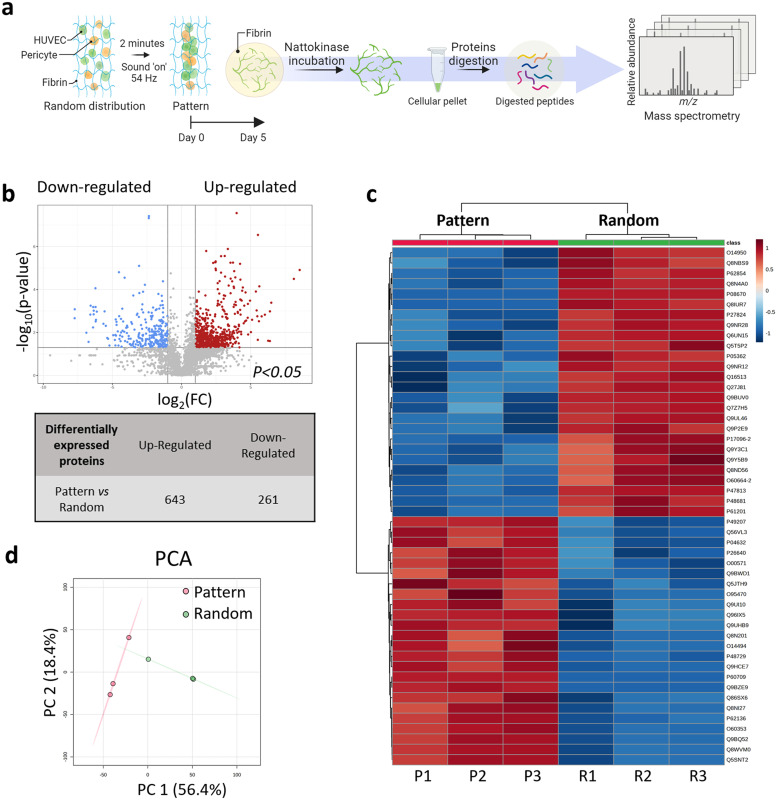
Untargeted differential proteomics analysis to investigate the impact of enhanced local cell density after sound patterning of centimeter-scale microcapillary networks. (a) At day 0, Green Fluorescent Protein-Expressing human umbilical vein endothelial cells (GFP-HUVEC) and human pericytes from placenta (hPC-PL) were collected and mixed at a ratio of 10:1 in a single-cell suspension made in fibrin, then dispensed into the sound patterning chamber with a round geometry. Before gelation, Faraday waves were generated at the liquid-air interface upon application of 54 Hz frequency vibration, and sound-induced hydrodynamic forces condensed the cells into reproducible pattern of 4 concentric circles in less than 2 min. At day 5, the concentric circles pattern samples were washed with PBS and incubated at 37 °C with 1 ml of a 2 mg mL^−1^ Nattokinase solution prepared in 1 mM EDTA PBS. Cellular material was collected in pellets, which underwent protein digestion. The digested peptides were then analyzed by mass spectrometry. (b) Proteomic analysis of the patterned capillaries after 5 days in culture revealed over 900 differentially expressed proteins compared to the stationary condition (random). 643 proteins were up-regulated and 261 down-regulated in the patterned microcapillaries compared to the random condition (*n* = 3). The heatmap (c) shows the 50 most significant proteins, and (d) the Principal Components Analysis (PCA) of the dataset reduced to two dimensions shows spatial separation between the random and patterned conditions.

**Fig. 4:**
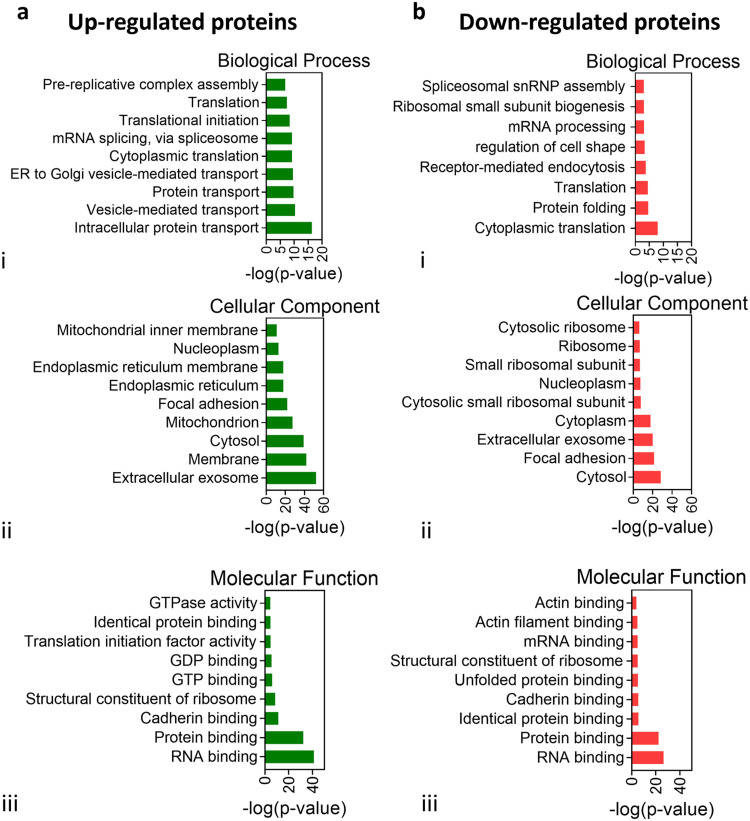
Gene Ontology (GO) enrichment analysis after proteins quantification on the concentric circles pattern. Top 9 significantly enriched GO (−log10 (*p*-value) >1.3) terms of the target genes in the cellular components, molecular function, and biological processes domains, either (a) up- or (b) down- regulated, resulted from DAVID software analysis. The complete list of GO annotation for the significantly differentially expressed associated genes is reported in supplementary information Fig. S5.

**Fig. 5:**
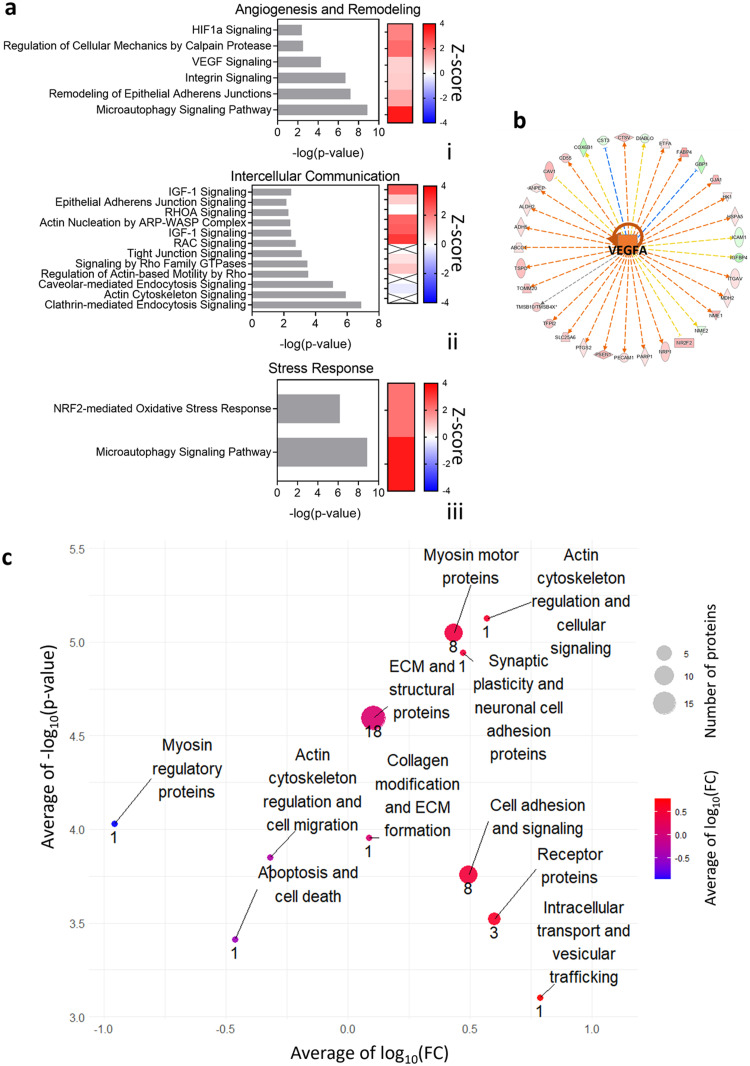
IPA analysis, upstream regulators, and proteins with altered expression grouped by function. (a) Ingenuity Pathway Analysis (IPA) identified the most representative altered canonical pathways targeted by the differentially expressed proteins in the concentric circles patterned microcapillaries, grouped by their involvement in Angiogenesis and Remodeling (i), Intercellular Communication (ii), and Stress Response (iii). (*p*-value <0.05). (b) VEGFA was identified as one of the most representative upstream regulators after IPA analysis. The complete list of identified significantly different canonical pathways is reported in supplementary material Excel file S1. In addition to the general predictions from DAVID and IPA analysis, the set of significant differentially expressed proteins was manually curated. The most relevant proteins, contextual to the experimental conditions, were clustered together according to their known functions and represented in the bubble plot. (c) The plot displays the average of log10(Fold Change) for the selected proteins, the average of −log2(*p*-value), and the number of proteins for each function group. A table with the IDs and names of the single proteins is reported in supplementary information Table S1.

After a 5 days cultivation, the capillary-like structures generated through sound patterning in concentric circles and those randomly assembled under static conditions underwent proteomic profiling (Fig. 3a), revealing different patterns. The hierarchical clustering heatmap (Fig. 3c) clearly showed the influence of the sound patterning in up- or down-regulating proteins’ expression, while principal component (PCA) analysis confirmed the presence of a proteomic signature associated with the patterned network (Fig. 3d). Among the 904 differentially expressed proteins, 643 were found to be up-regulated, while 261 showed down-regulation in the patterned capillary-like structures when compared to the randomly assembled counterparts (*n* = 3) (Fig. 3b). To further elucidate the functional implications of these differentially expressed proteins, associated genes were subjected to Gene Ontology (GO) enrichment analysis, categorizing them into biological processes (BP), cellular components (CC), and molecular functions (MF) (Fig. 4). This analysis suggested that the most significant upregulations were observed in the ``Intracellular Protein Transport'', ``Extracellular Exosome'', and ``RNA Binding'' categories within the BP, CC, and MF domains respectively (Fig. 4a, i,ii,iii). Additionally, Ingenuity Pathway Analysis (IPA) allowed the identification of the most altered canonical pathways correlated with the differentially expressed proteins in the patterned microcapillaries (Fig. 5a). IPA pathways were grouped based on their involvement in ``Angiogenesis and Remodeling'' (Fig. 5a, i), ``Intercellular Communication'' (Fig. 5a, ii), and ``Stress Response'' (Fig. 5a, iii). Within the ``Angiogenesis and Remodeling'' and the ``Stress Response'' categories, the ``Microautophagy Signaling Pathway'' emerged as the most significant activated pathway. Within the ``Intercellular Communication'', the ``Clathrin-mediated Endocytosis Signaling'' pathway, although being the most significantly different, could not be associated with a positive (>2) or negative (<−2) Z-score, which would indicate pathway activation or inhibition respectively. In this category, the ``RAC Signaling'' pathway was the most regulated of this category, as it showed the highest Z-score. Furthermore, the analysis of upstream regulators predicted the vascular endothelial growth factor A (VEGF-A) as one of the most representative proteins associated with the observed pro-vasculogenic pattern (Fig. 5b).

### 3.7 ECM and structural functions, cell motility, cell adhesion and signaling include the most abundant up-regulated proteins in sound patterned capillaries

In addition to the general predictions from DAVID and IPA analysis, the set of significant differentially expressed proteins was further analyzed. The most pertinent proteins were grouped based on their known functions, considering the experimental conditions (Fig. 5c and Table S1). Among the identified functional groups, the impact of cell condensation through sound patterning was highlighted in several categories.

Within the ECM and structural proteins group, 18 proteins were upregulated following cell condensation. This set included 11 Actin-related proteins, with the Beta-actin-like protein 2 (ACTBL2) showing the strongest up-regulation (log10(FC) = 3, *p*-value = 0.03). Collagen-related proteins were another subset in this category, with Collagen alpha-3(IV) chain (COL4A3) being the most up-regulated (log10(FC) = 0.554, *p*-value = 0.04). Furthermore, Matrix metalloproteinase-14 (MMP14) was also part of this functional group and up-regulated (log10(FC) = 0.529, *p*-value = 0.005).

In the capillary-like structures obtained after cell condensation, the functional group related to apoptosis and cell death revealed that the protein Caspase-6 (CASP6) was down-regulated (log10(FC) = −0.463, *p*-value = 0.033). It suggests that the cells not only did not activate programmed cell apoptosis pathways due to the mechanical movement imposed by the patterning, but also that condensation promotes cell survival compared to the static condition.

Furthermore, the cell adhesion and signaling functional group was the second most abundant enriched in differentially expressed proteins. Five integrin-related proteins were identified within this group: Disintegrin and metalloproteinase domain-containing protein 10 (ADAM10), Isoform 2 of Integrin alpha-3 (ITGA3), Platelet-derived growth factor receptor alpha (PDGFRA), Tyrosine-protein kinase receptor Tie-1 (TIE1), and Ephrin type-B receptor 2 (EPHB2). Among these, the latter exhibited the most significant up-regulation (log10(FC) = 0.742, *p*-value = 0.022), followed by PDGFRA (log10(FC) = 0.46, *p*-value = 0.029) and TIE1 (log10(FC) = 0.599, *p*-value = 0.04).

Lastly, the intracellular transport and vesicular trafficking category included the upregulated protein Trans-Golgi network integral membrane protein 2 (TGOLN2) (log10(FC) = 0.785, *p*-value = 0.044).

## 4 Discussion

This study explored the impact of cell density enhancement obtained through sound patterning on the proteomic profiles of in vitro biofabricated capillary-like structures. Sound patterning leverages sound frequencies below 200 Hz to transmit vertical vibration to a fluid containing particles within a macroscale container, inducing formation of Faradays waves. The hydrodynamic drag forces generated in the fluid can precisely condensate cells or cell aggregates within fluid phases. They are condensed beneath the nodal points of the standing waves where the resultant force acting on the particles is zero [23]. The deformation of the liquid-air interface, determined by a constant vertical vibration, can be mathematically described and the theoretical results can be compared with the experimental results for validating the observed physical behavior. In this study we used numerical simulations previously established [24,25,31,36], to validate the experimental results.

A unique feature of sound patterning is to create densely packed periodical cellular arrangements within ECM-like hydrogels [23,25,32]. We showed that the local cell density on the patterned lines had a 4-fold increase compared to stationary conditions, while keeping the initial cell seeding density constant. We showed that over a five-days culture period, the capillary-like structures maintained their pre-patterned morphologies after cell condensation. In fact, the radial intensity profile of the overview images of the networks presented a constant presence of peaks correlated with the patterned lines. In addition, the engineered constructs underwent cellular self-assembly in continuous capillary-like structures. The same cellular patterned were characterized and reported in previous studies from our group which demonstrated their maturation in vascular-like tissue with tubular 3D structure and hollow lumen formation [24,25]. Different pattern geometries resulted in distinct macroscopic orientations of the capillary-like structures, highlighting the versatility and precision of the sound patterning in controlling tissue organization. During the image analysis phase on the randomly seeded samples, the ImageJ function (OrientationJ) detected numerous small clusters of pixels correlated with the small capillary-like structures in the random sample image (Fig. S3a,iii). Instead, the function detected larger clusters of pixels with the same orientation from the samples obtained after sound patterning, which are correlated with the larger capillary-like structures formed in the latter case (Fig. S3a,i & a,ii). This discrepancy resulted in a higher count of orientation values in the random sample which we did not normalized in the orientation graph (Fig. 2g) since the cellular organizations are structurally different among the samples.

However, the molecular alterations of the cells imposed to the increased packing density remained an open question, especially regarding its implication in vascular engineering application.

Following cell condensation, self-assembly of the capillary-like structures and 5 days of tissue remodeling, in this work we characterized the proteomic profile of the formed capillary-like structures. We focused on the effect of the initially enhanced local cell concentration of the capillary-like structures on the proteomic signature. Although we detected that the cell condensation directly influenced the expression of over 900 proteins, further studies will need to clarify the effect on the proteomic signature of the sound vibration used for enhancing the local cell contraction. In fact, an appropriate control group of samples derived from highly concentrated cells, obtained with a different methodology, was not included in the current study. Nevertheless, we consider the sound vibration to have minimal direct impact on the cell biology since the application of the vibration occurs for a very short time (<2 min) and at low frequency (54 Hz).

As expected, the close cell-to-cell contact seems to increase the communication and signaling among the cells. In fact, proteins related to intracellular transport were found upregulated in the patterned samples, including several motor proteins, cytoskeleton regulatory proteins and intracellular transport proteins such as Myosin light chain 6B (MYL6B), Rho-related GTP-binding protein RhoB (RHOB) and Trans-Golgi network integral membrane protein 2 (TGOLN2). The latter may be involved in the release of extracellular vesicles, and accordingly several proteins within the “Extracellular Exosome” GO CC category were upregulated in the patterned capillaries. Therefore, their enhanced synthesis and secretion suggest for an increased crosstalk through exosomes between neighboring cells.

The protein expression was clearly impacted by the local cell condensation. Particularly, several RNA-binding proteins, known to be involved in the regulation of the gene expression at different stages of the protein synthesis, were upregulated, including RNA-binding motif protein (RBMX), single-stranded-interacting protein 1 (RBMS1), RNA transcription, translation, and transport factor protein (RTRAF).

Ingenuity Pathway Analysis was used to identify the pathways influenced by the local condensation of endothelial and pericytes cells on the capillary-like structures development, and in particular those linked to ``Angiogenesis and Remodeling''. Several pathways resulted activated after sound patterning, suggesting that tissue maturation and self-assembly was improved by the cellular condensation. Specifically, we found that the microautophagy signaling pathway was the most significantly activated. We can speculate that since the endothelial cells are close to each other, they tend to merge and fuse in order to form a larger capillary-like structure while recycling the excessive cellular material through autophagy mechanisms [37]. Moreover, autophagy has been reported playing a pivotal role in the prevention of mature vessels-related disease such as ischemic revascularization or atherosclerosis progression where such mechanism was found not properly controlled [38]. In this way cells could efficiently coordinate their assembly in a larger and longer capillary-like structures, similarly to the one reported in Fig. 2hii. We hypothesize that not all the cells present in the patterned line self-assemble and undergo tissue maturation, but some of them stop their development in favor of the greater capillary-like structures formation. We could obtain information also on the cell viability after cell condensation. Since one of the Caspase family proteins (CASP6) showed down-regulation, this could indicate a lower tendency in programmed cell death despite cells were imposed to a mechanical stress.

Neighboring cells are bounded to each other through junctions, networking their cytoskeletons [39]. The activation of the remodeling of epithelial adherens junctions pathway suggest that the adherent junctions between cells formed more extensively after cell condensation. Pointing towards the same direction, also the upregulation of the integrin signaling [40] suggest that cell-to-cell interactions by ECM deposition is increasing due to cellular condensation. This could be explained since the self-assembly of a larger capillary-like structure would require stronger interaction also between cells and as well as a higher ECM deposition to complete physiological tissue maturation.

Lastly, we detected an activation of the VEGF signaling pathway. VEGF is a key receptor protein involved in angiogenesis and vasculogenesis [41]. This could indicate either that the cellular condensation induces an overexpression of VEGF membrane receptors or that VEGF-A from pericytes is increased. Supporting the idea that cell condensation enhances the VEGF signaling, we also found upregulation in two important proteins related to this pathway, the vascular guidance receptor Neuropilin-1 (NRP1) and the Ephrin type-B receptor 2 (EPHB2). Furthermore, the analysis of upstream regulators identified VEGF-A as the most likely principal regulator of the detected molecular changes.

## 5 Conclusion

In the presented work, we applied sound patterning technology to enhance local cell density and for the biofabrication of capillary-like structures with on-demand morphologies. The fourfold enhancement of local cell density closely resembled the physiological cellular packing density in native tissue, leading to the formation of continuous capillary-like structures along the pattern line. Proteomics was used to elucidate the changes induced by enhancing local cell concentration within a natural extracellular matrix. The proteomic profile of sound-patterned microcapillary networks compared to randomly assembled ones demonstrated significant differences. Cellular communication, remodeling, and interaction with the extracellular matrix were among the processes modulated in response to cell condensation. Therefore, sound patterning could be implemented in future vascularized in vitro modeling of tissues characterized by varied capillary morphologies, such as the linear or twisty organization found in healthy or tumoral vasculature. This approach paves the way for fabricating in vitro biological constructs with diverse morphologies at high cell density, preserving tissue functionality and physiology.

## CRediT authorship contribution statement

**N. Di Marzio:** Methodology, Investigation, Data curation, Formal analysis, Visualization, Writing – original draft. **R. Tognato:** Methodology, Writing – original draft, Writing – review & editing. **E. Della Bella:** Supervision, Writing – review & editing. **V. De Giorgis:** Methodology, Writing – original draft. **M. Manfredi:** Methodology, Writing – original draft, Writing – review & editing, Conceptualization. **A. Cochis:** Conceptualization, Supervision. **M. Alini:** Funding acquisition, Supervision. **T. Serra:** Conceptualization, Funding acquisition, Supervision, Writing – review & editing.

## Declaration of competing interest

The authors declare that they have no known competing financial interests or personal relationships that could have appeared to influence the work reported in this paper.

## Data Availability

Data will be made available on request.

## Appendix Supplementary materials

Image, application 1

Image, application 2
