# Supplementary material for: Differential proteomics profile of microcapillary networks in response to sound pattern-driven local cell density enhancement
Source: Biomater Biosyst. 2024 Mar 29;14:100094. doi: 10.1016/j.bbiosy.2024.100094 (PMC11001772; doi:10.1016/j.bbiosy.2024.100094)
Supplement: Supplementary file 2 [file mmc2.docx]

Supplementary material

**Differential proteomics profile of microcapillary networks in response to sound pattern-driven local cell density enhancement**

N. Di Marzio^1,2^, R. Tognato^1^, E. Della Bella^1^, V. De Giorgis^3^, M. Manfredi^3^, A. Cochis^2^, M. Alini^1^, T. Serra^1,4^

1. AO Research Institute Davos, 7270 Davos, Switzerland
2. Department of Health Sciences, Center for Translational Research and Autoimmune and Allergic Diseases (CAAD), University of Piemonte Orientale, 28100 Novara, Italy

Università del Piemonte Orientale (UPO), 28100 Novara, Italy

1. Department of Translational Medicine, Center for Translational Research and Autoimmune and Allergic Diseases (CAAD), University of Piemonte Orientale, 28100 Novara, Italy

University of Piemonte Orientale, 28100 Novara, Italy

1. CTR Department, MERLN Institute for Technology-Inspired Regenerative Medicine, Maastricht University, 6229ET Maastricht, the Netherlands

Corresponding author: Tiziano Serra, [tiziano.serra@aofoundation.org](mailto:tiziano.serra@aofoundation.org). AO Research Institute Davos, Clavadelerstrasse 8, 7270, Davos Platz, Switzerland.

**
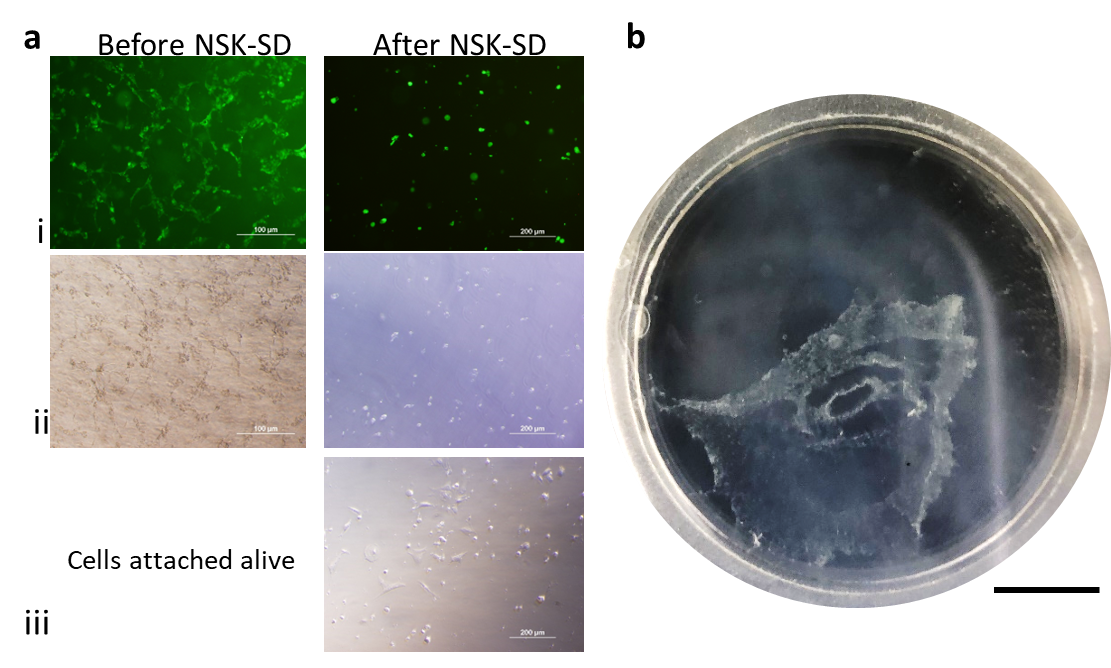
**

**Figure S1**. **Fibrin degradation after Nattokinase incubation.** The efficacy of Nattokinase (NSK-SD) in fibrin degradation while preserving cell viability was first test on self-assembled microcapillaries in in fibrin casted within a standard 96-well plate. The self-assembled capillary-like structures were imaged (a, i,ii) before NSK-SD incubation and (b, i,ii) after 20 min after incubation with 2 mg×mL^-1^ NSK-SD in PBS 1 mM EDTA at 37 °C, 5% CO_2_. The collected cells were seeded in a clean well and cultured with EGM-2 medium. 24 h after seeding, (b, iii) the collected cells were imaged and showed to be alive and attached to the culture plate. The 2 mg×mL^-1^ NSK-SD formulation was then used on the samples which underwent proteomic analysis. After 20 min incubation at 37 °C, 5% CO_2_, the cellular organization in concentric circles of the patterned samples could be observed after fibrin degradation. Scale bar = 5 mm.

**
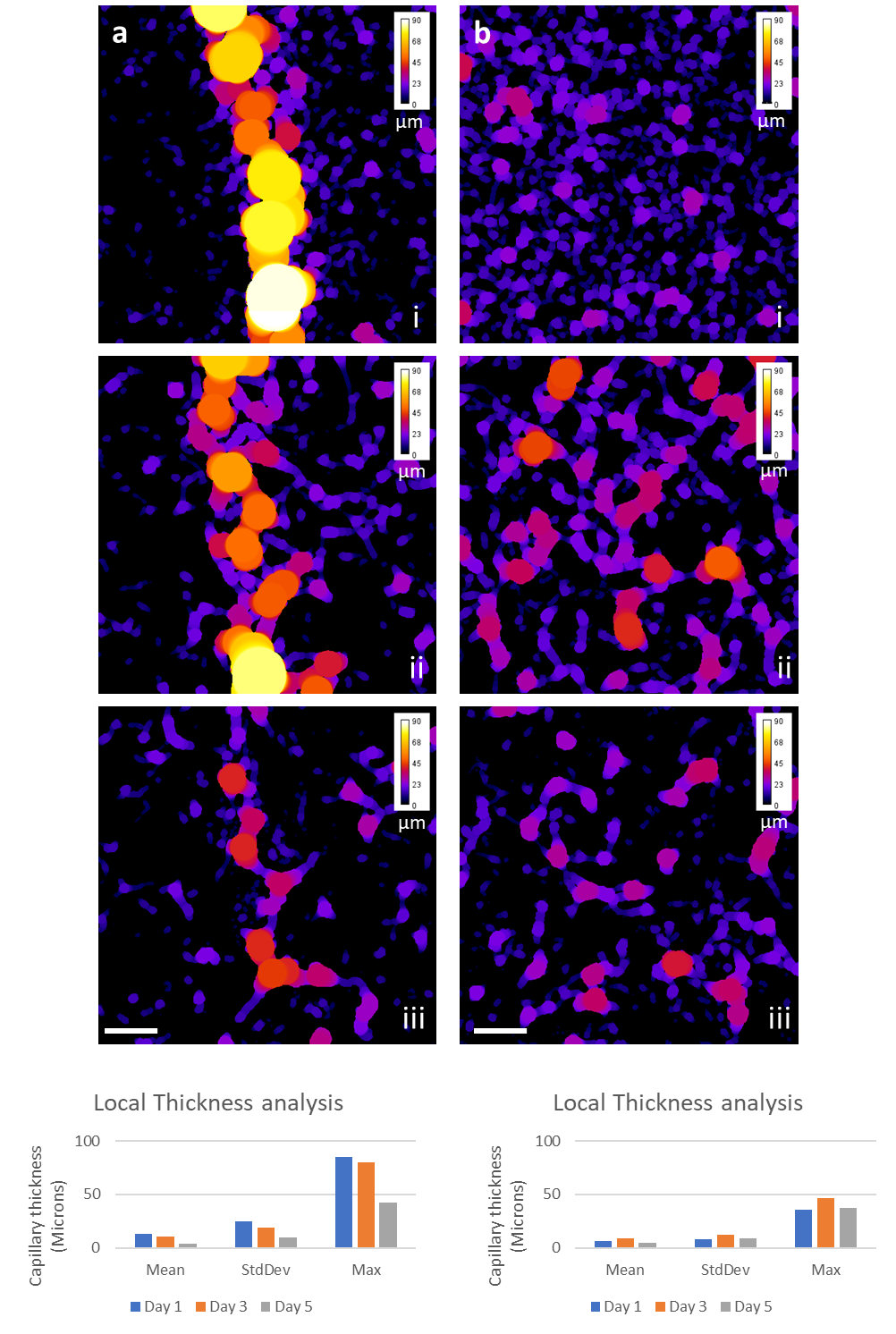
**

**Figure S2**. **Local thickness analysis at day 1,3,5.** Local thickness color map obtained from the patterned and random capillaries images (a, b) at day 1, 3, 5 (i, ii, iii). (c) the local thickness results for the patterned capillaries during the 5 days in culture were 13.1 ± 24.7 µm, 10.6 ± 18.9 µm, and 4.1 ± 9.5 µm, whereas 6.6 ± 7.8 µm, 9.3 ± 12.5 µm, and 4.5 ± 8.8 µm for the random distribution in stationary conditions, measured at day 1, 2, and 3, respectively (mean ± SD). The maximum thickness value measured for the patterned sample was 85 µm, 80 µm and 42 µm, whereas for the random sample 35 µm, 47 µm, 37 µm, at the same time points.


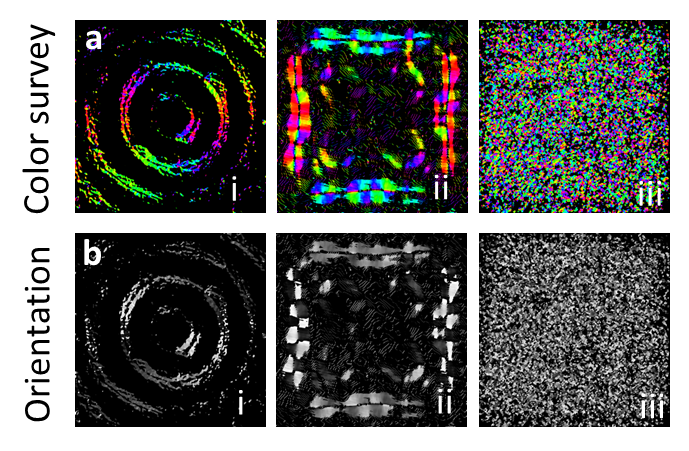


**Figure S3. Color coded angles survey and orientation maps obtained from the OrientationJ function.**


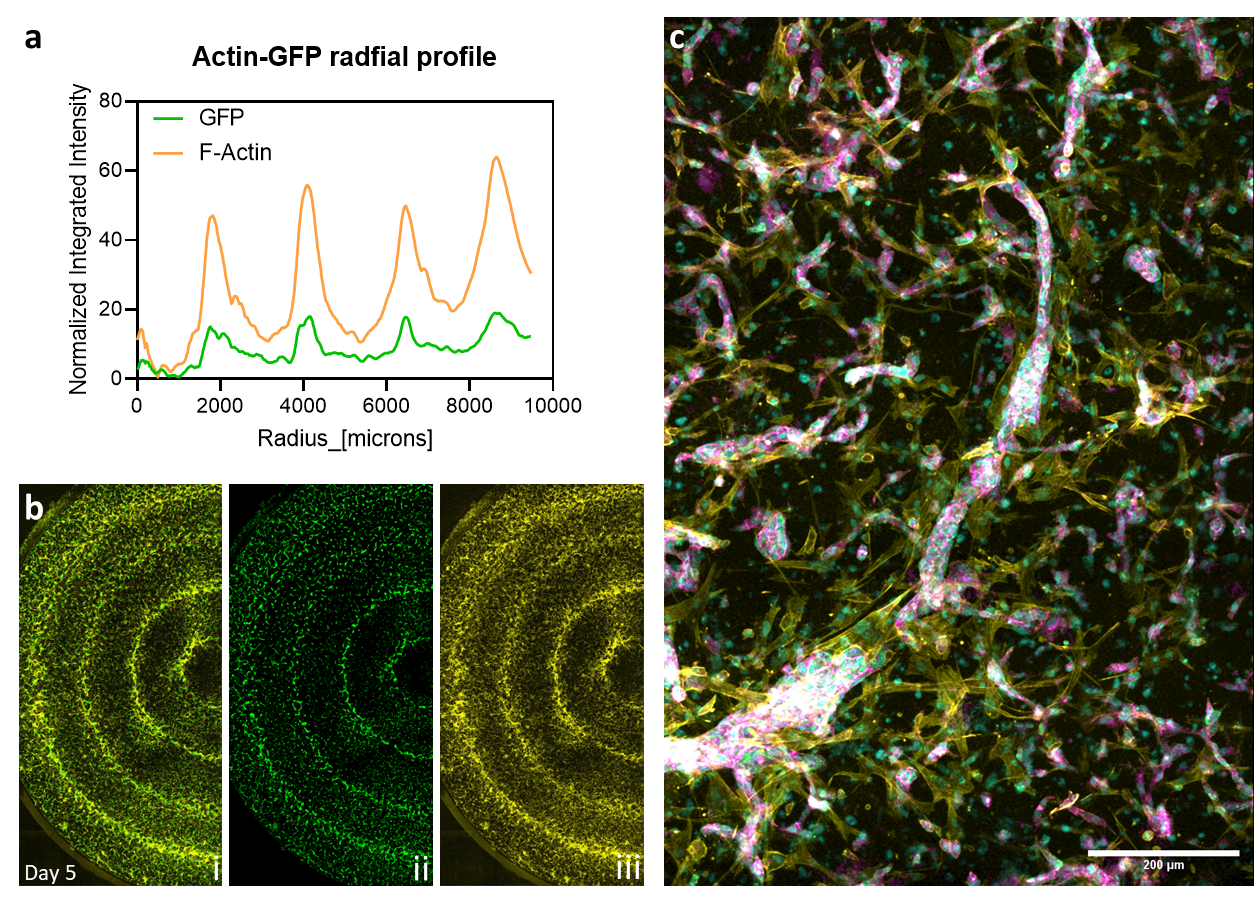


**Figure S4. Pericytes recruitment**

5 days after sound patterning, pericytes cells were found in the same spatial distribution as the capillary structures. Image analysis on the overview images after F-actin staining of the concentric microcapillary patterns showed wider intensity peaks corresponding to the pattern lines, suggesting increased pericytes density around the capillaries. Higer magnification microscopy confirmed pericyte proximity and alignment with the capillary structures (green = GFP-HUVEC, yellow = F-actin, violet = VE-cadherin)


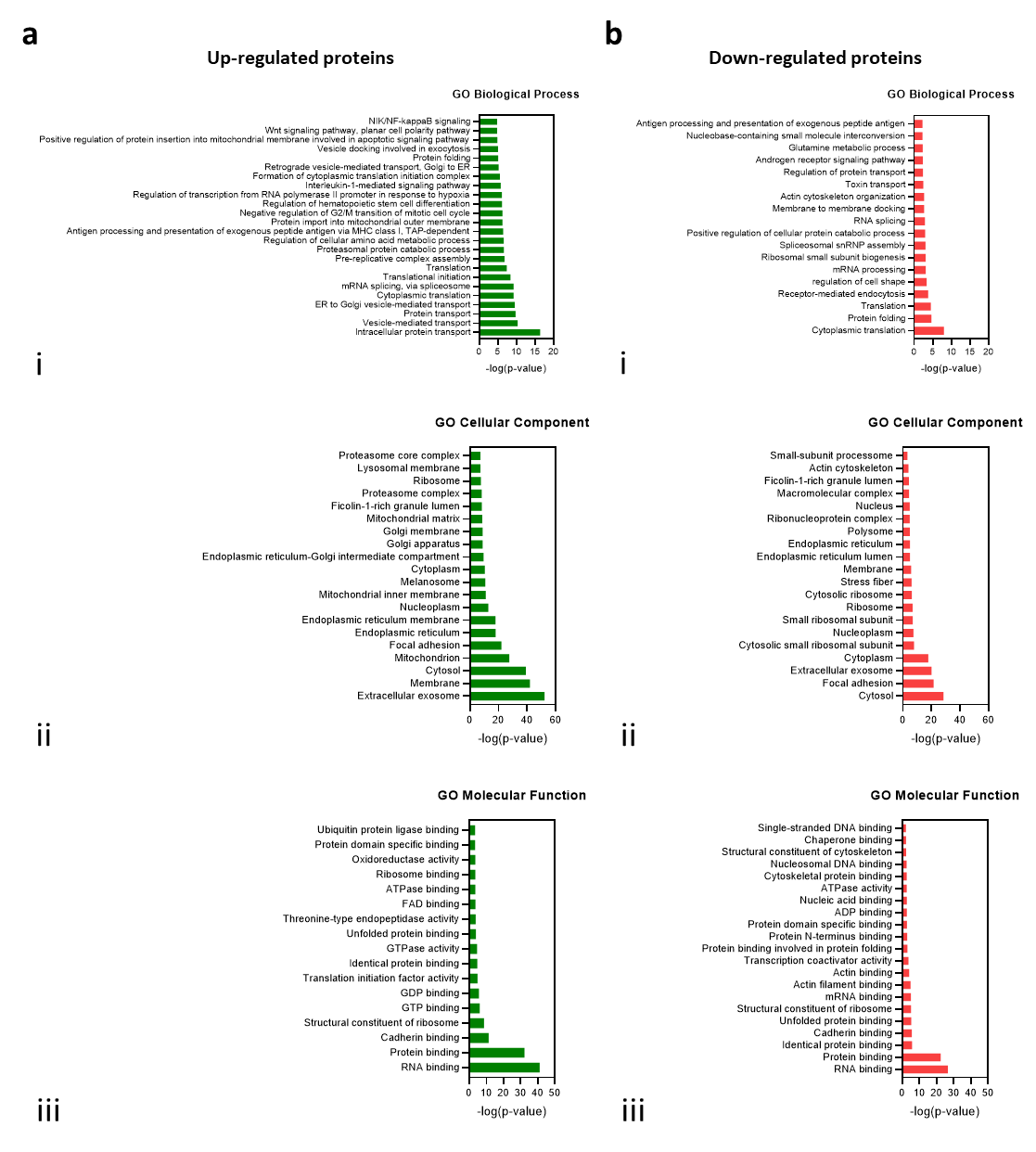


**Figure S5. Complete list of GO annotation for the significantly differentially expressed associated genes.**

**Table S1** Selected differentially expressed proteins grouped by function.
